# Late‐Stage Skeletal Muscle Transcriptome in Duchenne Muscular Dystrophy Shows a BMP4‐Induced Molecular Signature

**DOI:** 10.1002/jcsm.70005

**Published:** 2025-07-10

**Authors:** Hanna Sothers, Xianzhen Hu, David K. Crossman, Ying Si, Matthew S. Alexander, Merry‐Lynn N. McDonald, Peter H. King, Michael A. Lopez

**Affiliations:** ^1^ Division of Pulmonary, Allergy, and Critical Care Medicine, Department of Medicine University of Alabama at Birmingham (UAB) Birmingham Alabama USA; ^2^ Genetics, Genomics and Bioinformatics, Graduate School of Biomedical Sciences University of Alabama at Birmingham (UAB) Birmingham Alabama USA; ^3^ Department of Pediatrics, Division of Pediatric Neurology University of Alabama at Birmingham (UAB) Birmingham Alabama USA; ^4^ Children's of Alabama Birmingham Alabama USA; ^5^ Department of Genetics, School of Medicine University of Alabama at Birmingham (UAB) Birmingham Alabama USA; ^6^ Department of Neurology University of Alabama at Birmingham (UAB) Birmingham Alabama USA; ^7^ UAB Civitan International Research Center (CIRC) Birmingham Alabama USA; ^8^ UAB Center for Exercise Medicine Birmingham Alabama USA; ^9^ Birmingham Veterans Affairs Medical Center Birmingham Alabama USA; ^10^ Department of Epidemiology, School of Public Health University of Alabama at Birmingham (UAB) Birmingham Alabama USA; ^11^ Killion Center for Neurodegeneration and Experimental Therapeutics University of Alabama at Birmingham (UAB) Birmingham Alabama USA

**Keywords:** *BMP4*, Duchenne muscular dystrophy, *SMAD8*, *TGFβ1*

## Abstract

**Background:**

Duchenne muscular dystrophy (DMD) is a fatal X‐linked recessive disease due to loss‐of‐function variants in the *DYSTROPHIN* gene. DMD‐related skeletal muscle wasting is typified by an aberrant immune response involving upregulation of the TGFβ family of cytokines, like TGFβ1 and BMP4. We previously demonstrated that bone morphogenetic protein 4 (BMP4) is increased in DMD and BMP4 stimulation induces a 20‐fold upregulation of *Smad8* transcription in muscle cells. However, the role of BMP4 in late‐stage DMD skeletal muscle is unknown. We hypothesized that BMP4 signalling is a driver of aberrant gene expression in late‐stage human DMD skeletal muscle detectable by a transcriptomic signature.

**Methods:**

Transcriptomes from skeletal muscle biopsies of late‐stage DMD versus non‐DMD controls and C2C12 muscle cells with or without BMP4 stimulation were generated using RNA‐Seq. We tested transcriptional differences at the single transcript level in skeletal muscle biopsy samples from three patients with DMD and compared them to three non‐DMD. They were then analyzed by Ingenuity Pathway Analysis, weighted gene coexpression network analyses (WGCNA) and Gene Set Enrichment Analysis (GSEA). Key hub and high‐fold change genes overlapping in the DMD and BMP4 muscle transcriptomes were validated in additional primary and bulk skeletal muscle samples.

**Results:**

A total of 3048 transcripts in the human muscle and 5291 transcripts in C2C12 muscle cells were differentially expressed. WGCNA identified an overlapping molecular signature of 1027 genes dysregulated in DMD muscle that were induced in BMP4‐stimulated C2C12 muscle cells. *SERPING1* and *Aff3* were identified as the top hub genes. Highly upregulated DMD muscle transcripts that overlapped with BMP4‐stimulated C2C12 muscle cells included *ADAM12*, *SERPING1*, *SMAD8* and *SFRP4*. DMD skeletal muscle analysis showed aberrant upregulation of TGFβ signalling, extracellular matrix remodelling and collagen biosynthesis pathways, in contrast to inhibited mitochondrial and metabolic pathways.

**Conclusions:**

In summary, the DMD transcriptome was characterized by dysregulation of immune function, ECM remodelling and muscle bioenergetic metabolism. We additionally define a late‐stage DMD skeletal muscle transcriptome that overlaps with a BMP4‐induced molecular signature in C2C12 muscle cells. This supports BMP4/Smad8 pathway as a disease‐driving regulator of transcriptomic changes in late‐stage DMD skeletal muscle. Further exploration of this cross‐species transcriptomic signature may expand our understanding of the evolution of dystrophic signalling pathways and the associated gene networks, which could be evaluated for therapeutic development.

## 1 Introduction

Duchenne muscular dystrophy (DMD) is a fatal X‐linked recessive disease caused by loss of dystrophin expression [1]. This triggers a cascade of events starting with destabilization of the sarcolemmal membrane of skeletal muscle. The instability of the sarcolemmal membrane leads to chronic cycles of injury and repair, triggering a cascade of secondary disease‐driving processes. This invokes an immune response with upregulation of cytokines such as TGFβ1 and BMP4 [2]. As DMD progresses to late stage, defined by loss of ambulation, there is a conversion of skeletal muscle to fibroadipocytic tissue. This progresses to generalized paralysis, respiratory failure and dilated cardiomyopathy resulting in death by the third to fourth decade. Anti‐inflammatory therapies have been helpful but remain unsatisfactory due to side effects and modest impact on progression and survival. The large size of *DYSTROPHIN* (2.5 Mb) is a major barrier to restoring dystrophin expression [3]. Identification of additional disease‐driving pathways is a key area for developing targeted preclinical therapies.

TGFβ signalling via canonical TGFβ receptor ligands is well studied in DMD, but the role of bone morphogenetic protein (BMP) mediated pathology is underexplored [4, 5]. Receptor antagonism of BMP4 by overexpression of noggin improved fibrosis in a *mdx* mouse model and was associated with enhanced myogenic differentiation [6]. BMP4 has also been previously shown to be upregulated in primary DMD muscle cells [7]. We have shown that *BMP4* mRNA is highly upregulated in late‐stage DMD skeletal muscle in parallel with *SMAD8* (officially *SMAD9*), a TGFβ intracellular transcription factor [8]. We further showed that SMAD8 is strongly activated by BMP4 in C2C12 muscle cells and exerts a negative effect via SMAD8 on the myotube fusion index in association with a suppression of muscle‐enriched microRNAs: miR‐1, miR‐133a and miR‐133b [8].

We hypothesized that large changes in the muscle transcriptome would result from DMD disease given these impairments. However, investigation of transcriptional changes in late‐stage DMD muscle has been slowed by decreasing use of muscle biopsy and difficulty with animal models reproducing severe dystrophic muscle disease seen in humans. As such, prior studies have consisted of younger DMD muscles using array hybridization panels [9, 10, 11, 12, 13, 14].

To address these challenges, we generated RNA‐Seq transcriptomic data, followed by pathway and network analysis to characterize the late‐stage human DMD skeletal muscle transcriptome. We then compared late‐stage DMD muscle transcriptome to that of BMP4‐stimulated C2C12 muscle cells and found a substantial overlap. These findings support BMP4/Smad8 pathway as a disease‐driving signalling pathway evident in late‐stage DMD skeletal muscle.

## 2 Methods

### 2.1 Human Muscle Samples

All human muscle biopsies were collected from patients under an approved UAB Institutional Review Board protocol (IRB300002164). Three human non‐DMD muscle samples were selected from an archive of remnant muscle biopsy tissues in the UAB Division of Neuromuscular Disease as previously detailed [15]. DMD males ranged in age from 15 to 20 years. The DMD muscle specimens were taken from paraspinous muscle from nonambulatory males undergoing scoliosis surgery. All DMD paraspinous muscle specimens showed dystrophic pathology. Non‐DMD specimens showed no dystrophic pathology and ranged in age from 8 to 59 years. Non‐DMD diagnostic specimens were taken from the deltoid, tibialis anterior and quadriceps muscles. Five DMD (average age = 15 years) and seven normal (average age = 26 years) skeletal muscle specimens from male patients were utilized for validation by qPCR.

### 2.2 Ingenuity Pathway Analysis

Data were analysed with the use of Qiagen IPA (QIAGEN Inc., Redwood City, CA, https://digitalinsights.qiagen.com/IPA) as previously described [16, 17]. Canonical pathways (CP) analysis identified the pathways from the QIAGEN Ingenuity Pathway Analysis library of CPs that were most significant to the data set. The likelihood of CP activation or inhibition is calculated as a *Z* score, which is based on enrichment of genes overlapping with the Ingenuity Knowledge Database. A *p* value is based on enrichment of genes overlapping in the CP with Ingenuity Knowledge Database. Molecules from the data set that met FDR < 0.05 and fold change > |2.0| were considered for the analysis. IPA ‘Analysis Match’ was performed on these transcripts. Briefly, 200 000 OmicSoft datasets were compared to our transcriptomic dataset based on shared patterns of pathways and ‘analysis‐ready’ genes. The results were filtered for the following criteria: Project = ‘Human Disease’, Case Disease = ‘Duchenne or Becker’, Case Cell Type issue ≠ ‘Cell’, ‘myoblast’, ‘myotube’ or ‘myocyte’.

### 2.3 RNA Isolation and qPCR Analysis

Total RNA was isolated from muscle tissue or cells as previously described [8]. cDNA was synthesized using SuperScript IV First‐Strand Synthesis System (Invitrogen/Thermo Fisher Scientific, Waltham, MA, United States) using 1 μg of RNA, Taqman assay probes and primers (Applied Biosystems/Thermo Fisher Scientific, Waltham, MA, United States). TaqMan reactions were performed using TaqMan Gene Expression Master Mix (Applied Biosystems/Thermo Fisher Scientific, Waltham, MA, United States). Samples were run on a ViiA 7 Real‐Time PCR System (Applied Biosystems/Thermo Fisher Scientific, Waltham, MA, United States) in 384‐well plates. Relative expression values were calculated using the ΔΔCT method with normalization to the reference gene.

### 2.4 Transcriptomic Analysis

Weighted Gene Correlation Network Analysis (WGCNA) is a well‐established tool for creating networks of coexpressed biomarkers, such as RNA transcripts [18]. The networks are generated using an agnostic approach, allowing for a wider range of transcripts to be identified. It was also used to test for preservation of coexpression patterns between independent transcriptomics datasets, enabling validation of transcripts in disease processes.

The WGCNA R package was used to identify coexpressed RNA transcripts and assess module preservation of the DMD network within the C2C12 network. The DMD data were filtered for low counts (*n* < 10 reads for each sample) prior to normalization with DESeq2. The variance stabilized expression was used for WGCNA. Signed correlation networks were built using a biweight midcorrelation. Soft thresholding powers (*β*) of 10 for the DMD network and 16 for the C2C12 network were calculated using approximate scale‐free topology. WGCNA used hierarchical clustering to separate the transcripts into modules. Modules with ≥ 70% similarity were merged. The average expression profile of a module, eigengene, was used to assess the correlation of modules to the following traits: DMD status, age, sex, corticosteroid treatment and muscle pathology. Modules with a *p* value of < 0.05 were significantly correlated to a trait. The correlation of a gene's expression to a module's eigengene, *kME*, was used as a measure of module membership. A cutoff of *kME* > 0.8 was used to identify hub genes. The top hub genes for each module were selected using WGCNA's function ‘chooseTopHubInEachModule’.

Preservation of human genes in the C2C12 modules was carried out on a subset of transcripts with a one‐to‐one mapping between human and mouse Ensembl IDs [19]. Module preservation calculates a *Z*‐summary statistic where values > 2 indicate moderate preservation and > 10 indicate high preservation.

### 2.5 Network Analysis

The node and edge data from the resulting topological overlap adjacency matrices were generated using WGCNA. Edge weight represents correlation strength between two transcripts, and node size corresponds to the degree of connectivity (hubness) of an individual transcript to other transcripts in the network. Edges below a set cutoff weight were excluded. Cutoffs were selected based on the distribution of edge weights in the module. Cutoffs for individual modules were selected based on the edge weight distribution as follows: turquoise, 0.400 (human) and 0.441 (C2C12), and blue, 0.450 (human) and 0.387 (C2C12). Cytoscape (Version 3.10.0) was used to visualize the networks. In a network, first neighbours are nodes that share an edge. First neighbour networks were generated for relevant genes from the adjacency matrices from WGCNA. Genes with many connections were limited to the top 10–20 nodes with the highest edge weight. Top connected genes were filtered for LOG_2_FC > |1| and adjusted *p* value < 0.05 with edge weight > 0.45.

## 3 Results

### 3.1 Late‐Stage DMD Human Skeletal Muscle Transcriptome

**FIGURE 1:**
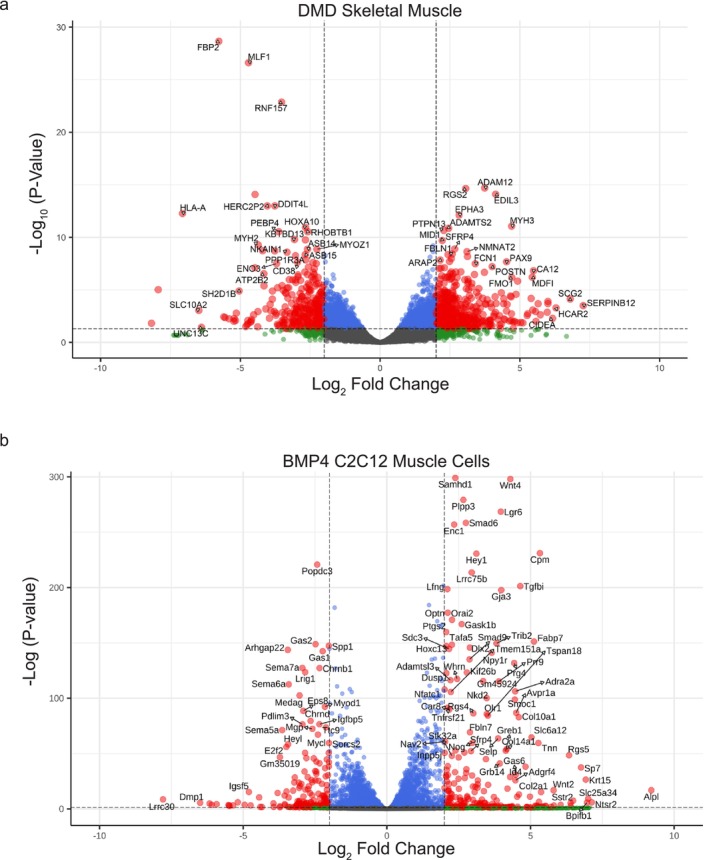
RNA‐seq analysis showing differential gene expression of DMD muscle and BMP4‐stimulated C2C12 muscle cells. (a) Volcano plot of DMD skeletal muscle transcriptome showing −log_10_ of adjusted *p* value versus log_2_‐fold change. Dashed vertical lines mark log_2_‐fold change > |2|. Dashed horizontal line marks adjusted *p* value < 0.05. Red indicates significant and > |2| log_2_‐fold change. Blue indicates significant but log_2_‐fold change < |2|. Green indicates not significant and log_2_‐fold change > |2|. Grey indicates not significant and log_2_‐fold change < |2|. (b) Volcano plot of BMP4‐stimulated C2C12 muscle cells (C2C12) transcriptome showing −log_10_ of adjusted *p* value versus. log_2_‐fold change. Dashed vertical lines mark log_2_‐fold change > |2|. Dashed horizontal line marks adjusted *p* value < 0.05. Red indicates significant and > |2|log_2_‐fold change. Blue indicates significant but log_2_‐fold change < |2|. Green indicates not significant and log_2_‐fold change > |2|. Grey indicates not significant and log_2_‐fold change < |2|.

Principal component (PC) analysis demonstrated stratification of the DMD from the non‐DMD muscles using the first two PCs (Figure S1). A total of 3048 transcripts were differentially expressed at a false discovery rate (FDR) < 0.05 (Table S1). Among these, 512 were upregulated and 285 were downregulated with log_2_ fold‐change (LOG_2_FC_DMD_) ≥ |2.0| (Figure 1a). The top transcripts upregulated in DMD muscle were *SERPINB12* [LOG_2_FC_DMD_ = 7.3 ± 1.61, FDR = 3.2 × 10^−04^], *SCG2* [LOG_2_FC_DMD_ = 6.8 ± 1.39, FDR = 7.6 × 10^−05^] and *HCAR2* [LOG_2_FC_DMD_ = 6.3 ± 1.44, FDR = 5.2 × 10^−04^]. *SERPINGB12* is part of the serine proteinase inhibitor (SERPIN) family. SCG2 is a member of the secretogranin family and is involved in cytokine/chemoattractant activity. *HCAR2*, which encodes the hydroxycarboxylic acid receptor 2, is a member of the G‐protein‐coupled receptor family [20]. *HLA‐A*, major histocompatibility complex (MHC), Class I, A, was one of the most downregulated [LOG_2_FC_DMD_ = −7.1 ± 0.86, FDR = 5.34 × 10^−13^].

Two‐thousand six‐hundred thirty‐four transcripts show a differential expression (LOG_2_FC > |1.0| and FDR < 0.05) in DMD muscle (Table S1). For example, TGFβ and immune‐related factors included upregulation of *TGFB1* [LOG_2_FC_DMD_ = 2.0 ± 0.42, FDR = 2.1 × 10^−04^]. *SMAD8* was the only *R‐SMAD* upregulated [LOG_2_FC_DMD_ = 1.9 ± 0.63, FDR = 2.09 × 10^−02^]. Myogenic regulatory factors were altered, including downregulation of *MEF2D* [LOG_2_FC_DMD_ = −1.1 ± 0.36, FDR = 2.15 × 10^−02^], downregulation of *MYF6* [LOG_2_FC_DMD_ = −1.4 ± 0.33, FDR = 7.56 × 10^−04^] and upregulation of *MYOG* [LOG_2_FC_DMD_ = 1.3, FDR = 5.11 × 10^−05^]. *UNC13C*, involved in neuromuscular junction function, was downregulated [LOG_2_FC_DMD_ = −6.4 ± 2.25, FDR = 3.67 × 10^−02^]. Markers of muscle regeneration, embryonic and foetal myosin heavy chain transcripts (*MYH3* and *MYH8*) were also upregulated. Further, several tissue and extracellular matrix (ECM) remodeling genes were dysregulated. A Disintegrin and Metalloprotease Domain (ADAM) metalloproteinases were broadly upregulated, including *ADAM12*, *ADAM22*, *ADAM28* and *ADAM* thrombospondins (*ADAMTS*)*2*, *ADAMTS3*, *ADAMTS7*, *ADAMTS12*, *ADAMTS15* and *ADAMTS16*. Similarly, matrix metalloproteinases (MMP) were broadly upregulated (*MMP2*, *MMP9*, *MMP14*, *MMP16* and *MMP19*) with *MMP9* being the highest [LOG_2_FC_DMD_ = 3.1 ± 1.03, FDR = 2.45 × 10^−02^]. *DMD* [LOG_2_FC_DMD_ = −2.0 ± 0.29, FDR = 5.67 × 10^−09^] and *CKM* [LOG_2_FC_DMD_ = −2.8 ± 0.58, FDR = 7.45 × 10^−05^] transcripts were significantly downregulated. These highest fold–changed muscle transcripts demonstrate evidence of dysregulation in TGFβ signalling, inflammation, fibrosis and myogenesis.

### 3.2 Ingenuity Pathway Analysis (IPA) of DMD Transcriptome

#### 3.2.1 Top Canonical Pathways and Regulators Dysregulated in DMD Muscle

**FIGURE 2:**
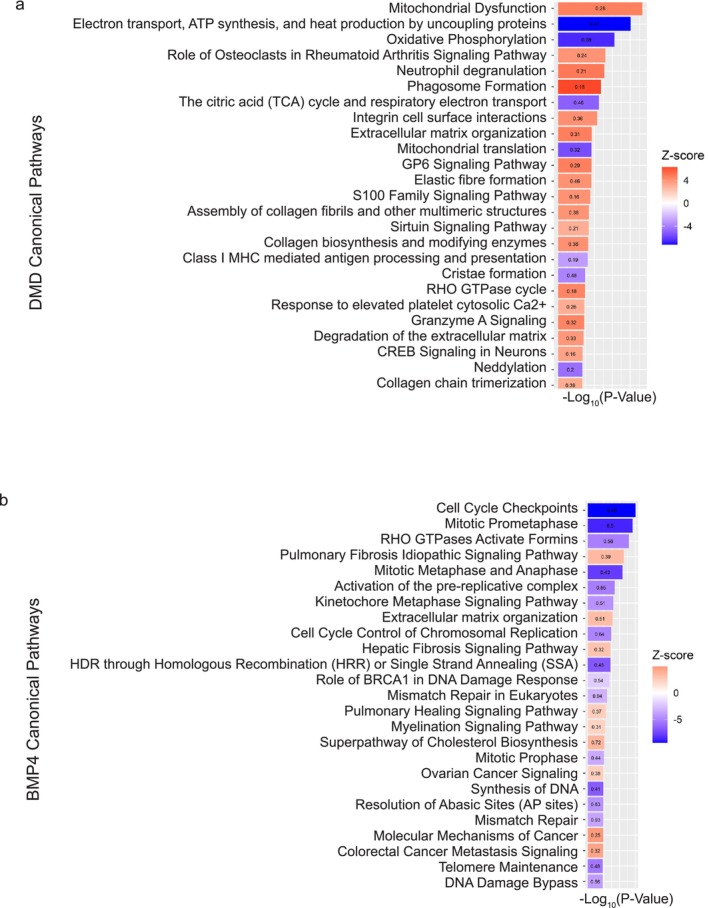
Ingenuity Pathway Analysis (IPA) predicted activated or inhibited canonical pathways. (a) DMD skeletal muscle IPA canonical pathways. Heat maps show *Z* scores for each canonical pathway ordered by −log_10_ of adjusted *p* value. The top 25 canonical pathways with *Z* scores > |3.0| were selected for comparison. Bars are labelled with the ratio of the number of overlapping genes within the canonical pathway. (b) BMP4‐stimulated C2C12 muscle cell canonical pathways predicted by IPA. Heat maps show *Z* scores for each canonical pathway ordered by −log_10_ of adjusted *p* value. The top 25 canonical pathways with *Z* scores >| 2| were selected for comparison. Bars are labelled with the ratio of the number of overlapping genes within the canonical pathway. *Z*‐score statistics indicate predicted state as activated (red) or inhibited (blue).

**FIGURE 3:**
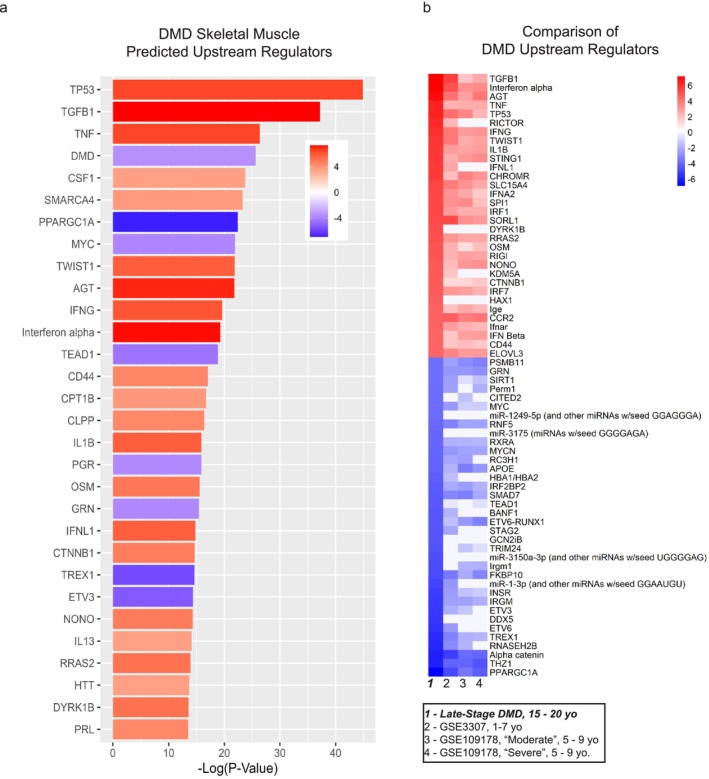
DMD skeletal muscle Ingenuity Pathway Analysis (IPA) predicted upstream regulators. (a) DMD upstream regulators are shown in heat maps with *Z* scores for each upstream regulator ordered by −log_10_ of adjusted *p* value. The top 30 upstream regulators with *Z* scores > |3.5| were selected for comparison. *Z*‐score statistics show predicted activated (red) or inhibited (blue) upstream regulators. (b) Heat map shows comparison of predicted upstream regulators from late‐stage DMD skeletal muscle transcriptome (1) with younger DMD muscle arrays that were identified by analysis match (2) GSE3307, (3) GSE109178/moderate and (4) GSE109178/severe. *Z*‐score statistics show predicted state as activated (red) or inhibited (blue).

The differentially expressed transcripts in the DMD case–control analysis (FDR < 0.05) encompassed several canonical pathways (CPs) (Figure 2a and Table S2). Activated CPs included ‘mitochondrial dysfunction,’ ‘role of osteoclasts in rheumatoid arthritis signalling,’ ‘neutrophil degranulation,’ ‘phagosome formation,’ ‘integrin cell surface interactions’ and ‘extracellular matrix organization.’ IPA also identified upstream regulators that were predicted to be activated or inhibited based on gene expression changes of transcriptional target molecules in each transcriptome (Table S3). There were 84 upstream regulators with predicted *Z* scores of more than |3.5|. TP53, TGFβ1, TNF and DMD were among the top 30 by *p* value (Figure 3a).

### 3.3 Comparison of Late‐Stage DMD Transcriptome to Public Transcriptomic Repositories

IPA ‘Analysis Match’ generated 14 dataset matches to our DMD transcriptome (Table S4). These dataset matches included data from previously deposited public DMD array dataset: GSE3307, GSE6011, GSE1004, GSE109178 and GSE13608. We also queried the NCBI GEO site for other high‐throughput sequencing profiles (Supporting Information S1).

GSE3307 were muscle biopsies from DMD males aged 5–9 years old with a dataset match (DM) *Z* score of 42. GSE109178 were muscle biopsies from DMD males aged 10 months old to 8 years old with a DM *Z* score of 36. Previously published DMD array‐based data (GSE3307 and GSE109178) showed similar upstream regulators predicted to be dysregulated. Our DMD data (DMD Non‐Amb) matched to datasets of dystrophic muscle from younger DMD patients (Figure 3b and Table S4) [14, 21]. Several cytokines were predicted to be activated upstream regulators including TGFβ1, TNF, IFNα/γ and IL1B. In our older DMD patient samples, TGFβ1 had higher predicted *Z*‐score activations (7.3) compared with GSE3307 (5.5) and GSE109178 (1.8 and 2.5). TP53, IFNα/γ, TNF and IL1B also showed higher levels of activation in the late‐stage DMD muscles. PPARGC1A, alpha catenin, CITED2 and miR‐1‐3p showed increased inhibition in the late‐stage DMD muscles in contrast to younger DMD samples. There were novel upstream regulators not predicted in the younger cohorts including DYRK1B, HAX1, miR‐1249‐5p, miR‐3175, GCN2iB, miR‐3150a‐3p and DDX5.

### 3.4 Weighted Gene Coexpression Network Analysis (WGCNA) of DMD Muscle Transcriptome

**FIGURE 4:**
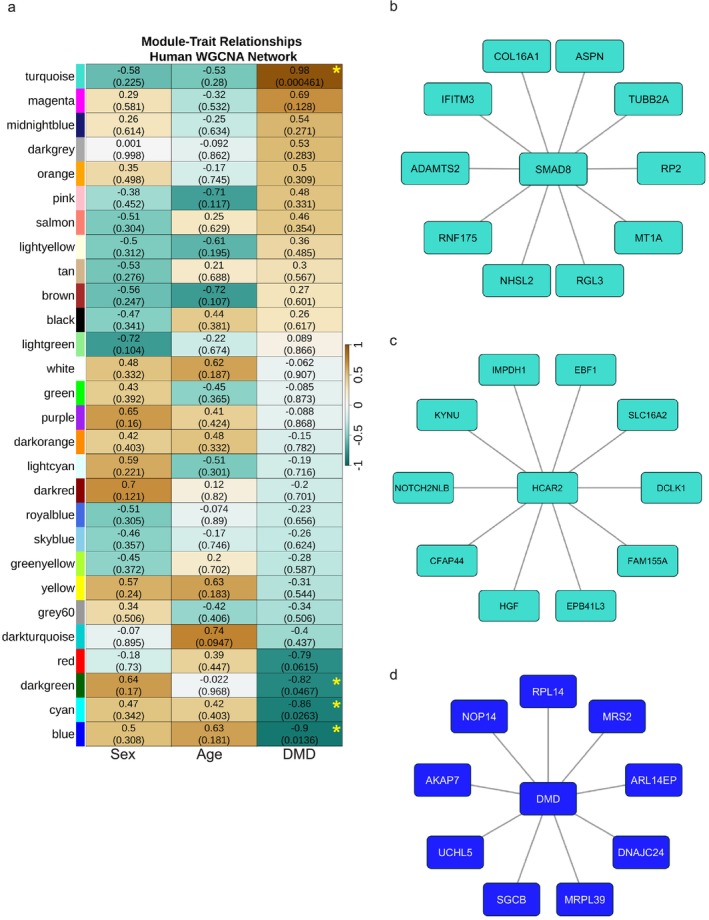
Weighted Gene Correlation Network Analysis of DMD muscle transcriptome. (a) Heat map of correlations between the module eigengenes and the DMD disease trait. Modules are labelled with *Z* score and (*p* value). Yellow * indicates *p* value < 0.05 for DMD trait. *Z* scores indicate upregulated (+) or downregulated (−) coexpression for each module. Colours are arbitrarily assigned. (b) Top connected genes coexpressed with *SMAD8* with edge weight (from adjacency matrix) ≥ 0.40 in the Turquoise_DMD_ (upregulated) module. (c) *HCAR2* coexpressed genes with edge weights (from adjacency matrix) ≥ 0.40. (d) Top 10 genes coexpressed with the DMD transcript in downregulated Blue_DMD_ module based on edge weight ≥ 0.45.

In the DMD muscle transcriptome, WGCNA identified a total of 27 modules of coexpressed genes (Table S5). Modules were evaluated for correlation to DMD status, age, sex, corticosteroid treatment and muscle pathology. A total of three modules were found to be significantly correlated with only DMD disease (turquoise [Turquoise_DMD_], blue [Blue_DMD_] and cyan [Cyan_DMD_]), and the dark green module was significantly correlated with both DMD disease status and muscle pathology (dark green_DMD_) (Figure 4a). The Turquoise_DMD_ module was comprised of 5730 genes collectively upregulated among DMD muscles with *SERPING1* as the top hub gene with a kME of 0.996 (Table S6). Top IPA upstream regulators in the Turquoise_DMD_ module included TGFβ1, IFNG, MMP9, IL1B and TP53. S*MAD8* was also a member of the Turquoise_DMD_ module. The Blue_DMD_ (4261 genes), Cyan_DMD_ (551 genes) and Dark green_DMD_ (401 genes) modules were downregulated in DMD muscle (Table S7). The Blue_DMD_ module, of which the DMD transcript (ENSG00000198947) is a member, was significantly downregulated (Table S7), and its top hub gene was *AKTIP*. Top IPA upstream regulators in the Blue_DMD_ module included PPARGC1A, BANF1 and DMD. *BMP4* was a member of the salmon module, which was not significantly associated with DMD disease state.

#### 3.4.1 Top Connected Genes

Sixteen genes showed the highest coexpression to *SMAD8* in the Turquoise_DMD_ module (Figure 4b and Table S8). *HCAR2*, the highest overall upregulated DMD transcript, showed a total of 47 genes with high connectivity (Figure 4c and Table S9). A total of 94 genes were connected to DMD in the significantly downregulated Blue_DMD_ module (Figure 4d and Table S10).

### 3.5 BMP4‐Stimulated Muscle Cell Transcriptome

Principal component analysis demonstrated wide separation of the BMP4‐stimulated **C2C12** muscle cells from vehicle treated cells (Figure S1b). In the BMP4‐induced transcriptome, 5291 transcripts were differentially expressed compared to vehicle (FDR < 0.05) (Table S11). Of these, 267 upregulated and 171 downregulated transcripts had LOG_2_FC_BMP4_ ≥ |2.0| (Figure 1b). Alkaline phosphatase, *Alpl*, was the highest upregulated transcript [LOG_2_FC_BMP4_ = 9.2 ± 1.04, FDR = 1.4 × 10^−17^]. Neurotensin receptor 2, *Ntsr2*, was the second highest upregulated transcript [LOG_2_FC_BMP4_ = 7.1 ± 1.34, FDR = 9.6 × 10^−07^]. It functions in apoptosis and cell viability. The most significantly downregulated genes included *Lrrc30* [LOG_2_FC_BMP4_ = −7.8 ± 1.22, FDR = 2.41 × 10^−09^]. Several downregulated genes were involved in cell cycle control (*Cdk6*, LOG_2_FC_BMP4_ = −1.5 ± 0.14, FDR = 3.78 × 10^−28^) or proliferation (*Top2a*, [LOG_2_FC_BMP4_ = −1.0 ± 0.08, FDR = 2.3 × 10^−38^]). *E2f2* [LOG_2_FC_BMP4_ = −3.5 ± 0.22, FDR = 1.0 × 10^−56^] was the most highly downregulated E2 transcription factor. The highest fold‐change transcripts dysregulated in BMP4‐stimulated C2C12 muscle cells are involved in bone abnormalities, hepatic cancer (ALPL), cell survival and cell cycle control.

### 3.6 Ingenuity Pathway Analysis (IPA) of BMP4‐Stimulated Muscle Cell Transcriptome

#### 3.6.1 Canonical Pathways in BMP4‐Stimulated C2C12 Muscle Cells

In BMP4‐stimulated C2C12 muscle cells, there were several CPs predicted to be activated or inhibited by BMP4 stimulation (Figure 2b and Table S12). The activated CPs included ‘pulmonary fibrosis idiopathic signalling pathway,’ ‘ECM organization’ and ‘HDR through homologous recombination or single strand annealing.’ The inhibited CPs included ‘cell cycle checkpoint’ (CCC), ‘mitotic prometaphase,’ ‘RHO GTPases activate formins,’ and ‘mitotic metaphase and anaphase.’ The CCC, for example, showed downregulation of numerous genes such as cyclin dependent kinases, cyclin dependent kinase inhibitors and tumor suppressor genes (*CDK1*, *CKD2*, *CDKN1A* and *TP53*).

#### 3.6.2 Predicted Upstream Regulators in BMP4‐Stimulated C2C12 Muscle Cells

In BMP4‐stimulated C2C12 muscle cells, the top three predicted activated protein‐encoding regulators were TGFβ1, XBP1 and TP53 (Table S13). The top predicted activated regulators among growth factors were Bmp2, Bmp4, Bmp10, Gdf2, Tgfβ1, Tgfβ2 and Tgfβ3. The only inhibited growth factors were Areg, Nog, Hgf and Angptl3. Among cytokines, Il1b, Il6, Prl and Wnt3a had the highest *Z* scores.

#### 3.6.3 Common Predicted Upstream Regulators in DMD and BMP4‐Stimulated C2C12 Muscle Cells

There were 188 common upstream regulators predicted to be activated or inhibited with a *p* value of overlap < 0.05 and predicted *Z* score > |2| in both DMD muscle and BMP4‐induced transcriptomes (Tables S3 and S13). For example, the top cytokine molecules included activation of TNF, IL1B, PRL, CSF2, IL6, IL13, IFNB1 and WNT3A. Similarly, top growth factors included activation of TGFβ1, AGT, GDF2, BMP10, PDGFC and TGFβ3. The top 5 microRNA upstream regulators were predicted to be inhibited, including miR‐1‐3p, miR‐3150a‐3p, miR‐1345‐5p, miR‐3175 and miR‐6825‐5p.

#### 3.6.4 Common DMD and BMP4 Canonical Pathways

There were 144 common CPs that were predicted to be activated or inhibited in both DMD muscle and BMP4‐stimulated transcriptomes with *p* value < 0.05 and *Z* score > |2| (Tables S2 and S12 and Figure 2a,b). The overall top 5 commonly activated CPs, by average *Z* score of two transcriptomes, were ‘molecular mechanisms of cancer’, ‘extracellular matrix organization’, ‘collagen biosynthesis’, ‘pulmonary fibrosis idiopathic signalling pathway’ and ‘role of osteoclasts in rheumatoid arthritis signalling pathway’. Similarly, the overall top 5 commonly inhibited CPs were ‘deubiquitination’, ‘striated muscle contraction’, ‘rhogdi signalling’, ‘glucose metabolism’ and ‘sulphur amino acid metabolism’.

### 3.7 Network Analysis of BMP4‐Stimulated Myoblast Transcriptome

WGCNA identified 22 modules of coexpressed genes in the C2C12 muscle cell transcriptome (Table S14).

**FIGURE 5:**
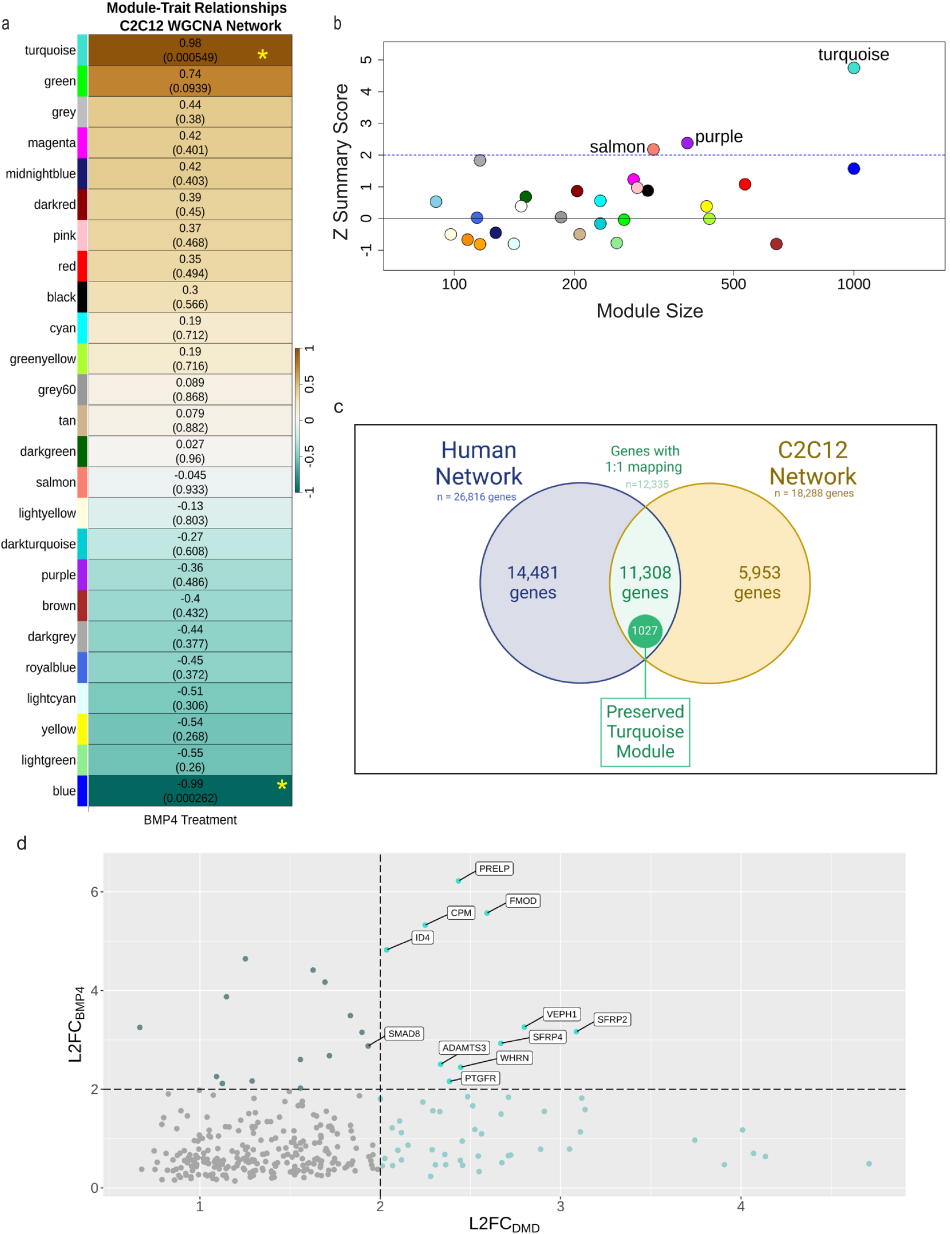
Weighted Gene Correlation Network Analysis of BMP4‐stimulated C2C12 transcriptome and comparison to DMD transcriptome. (a) Heat map showing correlation between the module eigengenes and BMP4‐stimulated C2C12 muscle cells. Modules are labelled with *Z* score and *p* value. Yellow * indicates *p* value < 0.05. *Z* scores indicate upregulated (+) or downregulated (−) coexpression for each module. Colours are arbitrarily assigned. (b) Module preservation analysis between the DMD and BMP4‐stimulated C2C12 muscle cells showing *Z*‐summary preservation statistic for each module and module size. The Turquoise_preserved_ module was moderately preserved with a *Z*‐summary score of 4.7. (c) Venn diagram of overlapping genes in the Turquoise_preserved_ module. A total of 12 335 genes showed a 1‐to‐1 mapping between the human and C2C12 networks of which 1027 were in the Turquoise_preserved_ module. (d) Plot of the log_2_ fold change from the DMD muscle transcriptome (LOG_2_FC_DMD_) and the BMP4‐simulated C2C12 transcriptome (LOG_2_FC_BMP4_) for genes present in the Turquoise_preserved_ module with an FDR < 0.05. Dashed lines indicate transcripts with LOG_2_FC > 2. Selected transcripts are labelled demonstrating high LOG_2_FC.

The Turquoise_BMP4_ (4615 transcripts upregulated) and Blue_BMP4_ (4452 transcripts downregulated) modules were identified as significantly correlated with BMP4 stimulation (Figure 5a and Tables S15 and S16). *Angptl2* and *Rfc3* were the associated hub genes for Turquoise_BMP4_ and Blue_BMP4_ modules, respectively.

### 3.8 Module Preservation Analysis Between DMD Muscle and BMP4‐Stimulated C2C12 Muscle Cells

Module preservation analysis tested whether the BMP4‐induced C2C12 muscle cell transcriptome had any overlapping module with the DMD muscle transcriptomic network (Table S17). We found moderate module preservation of the Turquoise_preserved_ module consisting of 1027 upregulated transcripts with a *Z*‐summary score of 4.7 (Figure 5b,c and Table S18). The preserved genes with the highest kMEs in the human and C2C12 networks, respectively, were *SERPING1* and *Aff3*. The highest upregulated transcripts in human DMD and BMP4‐induced transcriptomes are plotted in Figure 5d. S*MAD8* was also the only *SMAD* in the Turquoise_preserved_ module. No downregulated modules were significantly preserved. *UNC13C* [LOG_2_FC_DMD_ = −6.39 ± 2.25, FDR = 3.67 × 10^−02^; LOG_2_FC_BMP4_ = −2.99 ± 0.85, FDR = 2.0 × 10^−03^] and *ANO5* [LOG_2_FC_DMD_ = −2.1 ± 0.4, FDR = 1.3 × 10^−05^; LOG_2_FC_BMP4_ = −2.7 ± 0.3 FDR = 4.7 × 10^−15^] were the most downregulated transcripts in both transcriptomes.

Gene Set Enrichment Analysis of the Turquoise_preserved_ module identified significant human genes that were over‐represented (Table S19 and Figure S3). ‘Hallmark Epithelial Mesenchymal Transition’ was the top significant gene set in Hallmark collection. These genes define epithelial‐mesenchymal transition in wound healing, fibrosis and metastasis.

### 3.9 Validation of Overlapping Targets in DMD and BMP4‐Muscles by qPCR

#### 3.9.1 BMP4‐Stimulated C2C12 Muscle Cell Targets

**FIGURE 6:**
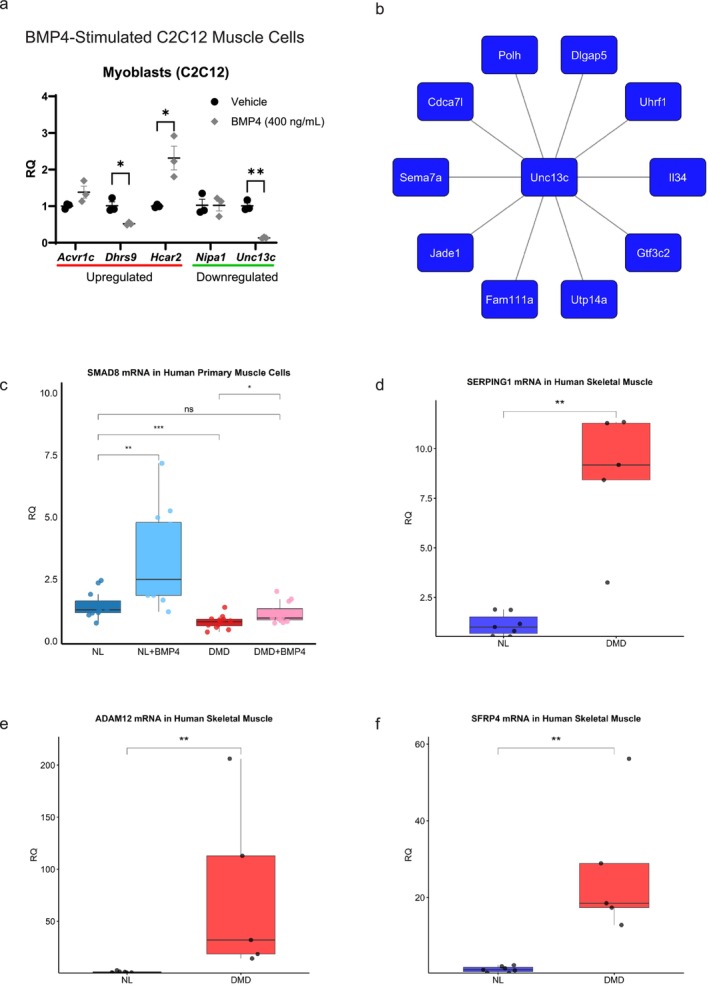
DMD dysregulated mRNA targets regulated by BMP4. (a) Plot shows mRNA expression levels (RQ) of *Acvr1c*, *Dhrs9*, *Hcar2*, *Nipa1* and *Unc13c* after BMP4 stimulation of C2C12 muscle cell as measured by quantitative RT‐PCR (qPCR). Bars show mean ± SEM of three biological replicates per group. *p* values compared to vehicle *< 0.05 and **< 0.01. Transcripts underlined in red and blue note their upregulation or downregulation, respectively, in the DMD transcriptome. (b) *Unc13c* coexpressed genes with edge weight (from adjacency matrix) ≥ 50th percentile (0.387) of adjacency matrix values. (c) *SMAD8* mRNA is increased by BMP4 in both normal (*N* = 3 cell lines) and DMD (*N* = 3 cell lines) primary skeletal muscle cells in growth conditions. Experiments repeated in triplicate. (d–f) *SERPING1*, *ADMA12* and *SFRP4* mRNA are increased in human DMD skeletal muscle compared to normal muscle (NL). RQ, relative quantity, reference genes: (a) *Gapdh* and (c–f) *RPS9*. Box and whisker plots show median line, interquartile range (IQR) and whiskers extending to 1.5 IQR. *p* values: *< 0.05, **< 0.01, ***< 0.001 and ****< 0.0001. A *t* test is used for (a) and Wilcoxon test is performed for (c)–(f).

In BMP4‐stimulated C2C12 muscle cells, we assessed several transcripts that were found to be highly dysregulated in the late‐stage DMD muscle, including *HCAR2* and *UNC13C* (Figure 6a). *Dhrs9* was downregulated by BMP4 stimulation in contrast to its upregulation in DMD muscle. *Hcar2*, which was the most upregulated late‐stage DMD muscle transcript, was induced more than twofold following BMP4 stimulation of C2C12 muscle cells. *Unc13c*, one of the most downregulated transcripts in DMD muscle, was suppressed by more than eightfold in BMP4‐stimulated cells. The top 10 *Unc13c* connected transcripts in BMP4‐stimulated muscle cells by WGCNA are shown (Figure 6b and Table S20).

#### 3.9.2 Human DMD Primary Muscle Cell and Skeletal Muscle Tissue

We next assessed if Smad8 was also dysregulated in DMD human primary muscle cells. We found that BMP4 upregulated *SMAD8* mRNA in both normal and DMD primary muscle cells (Figure 6c). The reduced level of *SMAD8* mRNA expression in the DMD primary muscle cells indicates that the tissue microenvironment is a crucial factor for the upregulation of *SMAD8* in DMD skeletal muscle.

We next evaluated *SERPING1*, *ADAM12* and *SFRP4* in human DMD and normal skeletal muscles using qPCR (Figure 6d–f). *SERPING1* was identified as a key hub gene of the Turquoise_preserved_ module, whereas *ADAM12* [LOG_2_FC_DMD_ = 3.7 ± 0.42, FDR = 2.0 × 10^−15^] and *SFRP4* [LOG_2_FC_DMD_ = 2.7 ± 0.38, FDR = 1.3 × 10^−09^] were high‐fold change and high‐significance transcripts of the Turquoise_preserved_ module. DMD skeletal muscle showed *SERPING1* was increased by a mean of 8.7‐fold compared to normal muscle. *ADAM12* increased by 77‐fold and *SFRP4* increased by 27‐fold compared to normal skeletal muscles. These data suggest that upregulation of BMP4 is one mechanism by which *Smad8*, *ADAM12*, *SFRP4* and *SERPING1* are upregulated in DMD skeletal muscles.

## 4 Discussion

We and others have previously found upregulation of *BMP4* and *SMAD8* mRNA in DMD [6, 8]. We demonstrated a role for BMP4 by showing it activates *SMAD8* and represses miR‐1, miR‐133a and miR‐133b [8, 15]. We hypothesized that BMP4/SMAD8 signalling is part of a disease‐driving pathway in DMD. To study this, we utilized RNA‐Seq on late‐stage DMD skeletal muscle and analysed the transcriptomic changes using network and pathway analysis. We identified three major processes in DMD skeletal muscle: (1) immune response dysfunction, (2) ECM remodelling and (3) myogenic/metabolic dysfunction. We additionally discovered a unique transcriptomic signature that is induced by BMP4 and overlaps with the DMD late‐stage skeletal muscle.

First, immune response dysfunction was characterized by upregulation of TGFβ signalling as seen by increased *TGFβ1*, *TGFβ3* and *SMAD8*, the canonical BMP downstream signal transducer. Our previous finding that all three isoforms of TGF*β* induce and activate Smad8 raises the possibility of cross‐activation of TGFβ1 downstream signalling, potentially mediated through noncanonical pathways [22]. This interconnectivity would have implications for therapeutic development given the evidence of TGFβ signalling redundancy.

*SERPING1* was highly upregulated and identified as a hub gene in the DMD and BMP4‐stimulated C2C12 muscle cell transcriptomes. *SERPING1* is involved in complement activation and acute phase response canonical pathways (CP). The acute phase response CP was predicted to be activated in both transcriptomes and implies ongoing proinflammatory activity mediated by BMP4. *SERPING1* encodes a C1 esterase inhibitor of complement activation (C1‐INH) and functions in suppressing inflammation. It was previously identified as a hub gene in a microarray database of DMD muscle samples (GSE6011) [9]. The upregulation of this hub gene in late‐stage DMD muscle suggests an ongoing role of complement activation at this stage. We newly identify *HCAR2* as the most upregulated transcript in the late‐stage DMD muscle (7.2‐fold). HCAR2 is recognized as a repressor of inflammation, and it is upregulated by TGFβ1 [23, 24]. In prior microarray studies from 5‐ to 9‐year‐old DMD males (GSE3307), *HCAR2* was minimally increased, suggesting it could serve as a novel biomarker for DMD progression. The anti‐inflammatory effect of HCAR2 was previously demonstrated in several models including DMD primary myoblasts involving IL‐6, in the *mdx* mouse, and in macrophages via nicotinic acid and NFκB suppression [23, 24, 25].

In contrast, we observed that *HLA‐A* is the most downregulated transcript. It is a major histocompatibility complex, Class I, A molecule ubiquitously expressed and functioning in antigen presentation to the immune system. A prior array study reports an upregulation of HLA genes, including *HLA‐A*, in association with younger DMD biceps brachii muscles [26]. *HLA‐A* downregulation in our samples suggests that attenuation of HLA genes is a marker of disease progression. However, we did not find that BMP4 reduced HLA gene expression. This suggests other pathways may drive suppression of HLA gene expression. Further evidence of proinflammatory and profibrotic responses in late‐stage DMD muscle was indicated by IPA‐identified activation of the ‘phagosome formation’ CP and several upstream regulators, notably TGFβ1, IFNα/γ and TNF group (Figures 2a and 3). Taken together, immune‐mediated mechanisms remain highly relevant in late‐stage DMD.

Second, ECM pathways were dysregulated with evidence of increased remodelling, matrix metalloprotease (MMP) activity and collagen deposition in late‐stage DMD muscle. *ADAM12* was one of the highest upregulated DMD muscle transcripts, and it was also strongly upregulated by BMP4 stimulation. *ADAM12* is linked to ‘inhibition of matrix metalloproteases’ and ‘ECM organization’ CPs. Both CPs were commonly and strongly predicted to be inhibited and activated, respectively. The ADAM/ADAMTS family functions in ECM organization and collagen biosynthesis. Its upregulation promotes muscle fibre loss, fibrosis and adipogenesis in *mdx* mice [27, 28]. The ‘inhibition of matrix metalloproteases’ was predicted to be inhibited, and correspondingly, several MMPs were highly upregulated including *MMP9*. MMP9 contributes to myofiber injury and fibrosis and impairs regeneration through its degradation activity on the basement and sarcolemmal membranes [29]. Prior array databases and other reports did not identify MMP9 as upregulated or active in younger DMD patient muscles (Figure 3b) [30, 31]. Increased MMP9 in late‐stage DMD muscle suggests that it is a marker of disease progression. Serum MMP9 has been identified as a potential biomarker of clinical progression with significantly higher levels detected in older DMD patients [32]. MMP9 also promotes TGFβ signalling by converting TGFβ to an active form via proteolytic processing. MMP9 knockout studies suggest a disease‐mitigating role for MMP9 in the late dystrophic stage in contrast to earlier stages [33].

Third, we found pathways related to myogenesis and loss of muscle metabolic bioenergetic functions. The top IPA identified CPs and upstream regulators pertinent to muscle loss included activation of ‘mitochondrial dysfunction’ and inhibition of metabolic categories (‘electron transport, oxidative phosphorylation and citric acid’). The ‘mitochondrial dysfunction’ CP was previously reported to be activated in younger DMD skeletal muscle microarrays (GSE3307), although with lower activation *Z* scores than our late‐stage DMD suggesting worsening of mitochondrial dysfunction with increasing age [14, 21]. These patterns reflect loss of normal metabolic activity with concomitant activation of mitochondrial dysfunction (Figure 2a–b).

*UNC13C* was one of the most downregulated transcripts in DMD muscle and BMP4‐stimulation of C2C12 muscle cells strongly reproduced this suppression. This suggests that BMP4 signalling is a strong inhibitor of *UNC13C* expression in C2C12 muscle cells. *UNC13C* function is not well explored, but prior reports in *C. elegans* show *unc* mutations confer severe impairments in motility and aberrant neuromuscular junction morphometry [34, 35]. *Unc13c* is also implicated in the promotion of myoblast differentiation and negatively regulated by TNFα [36].

**FIGURE 7:**
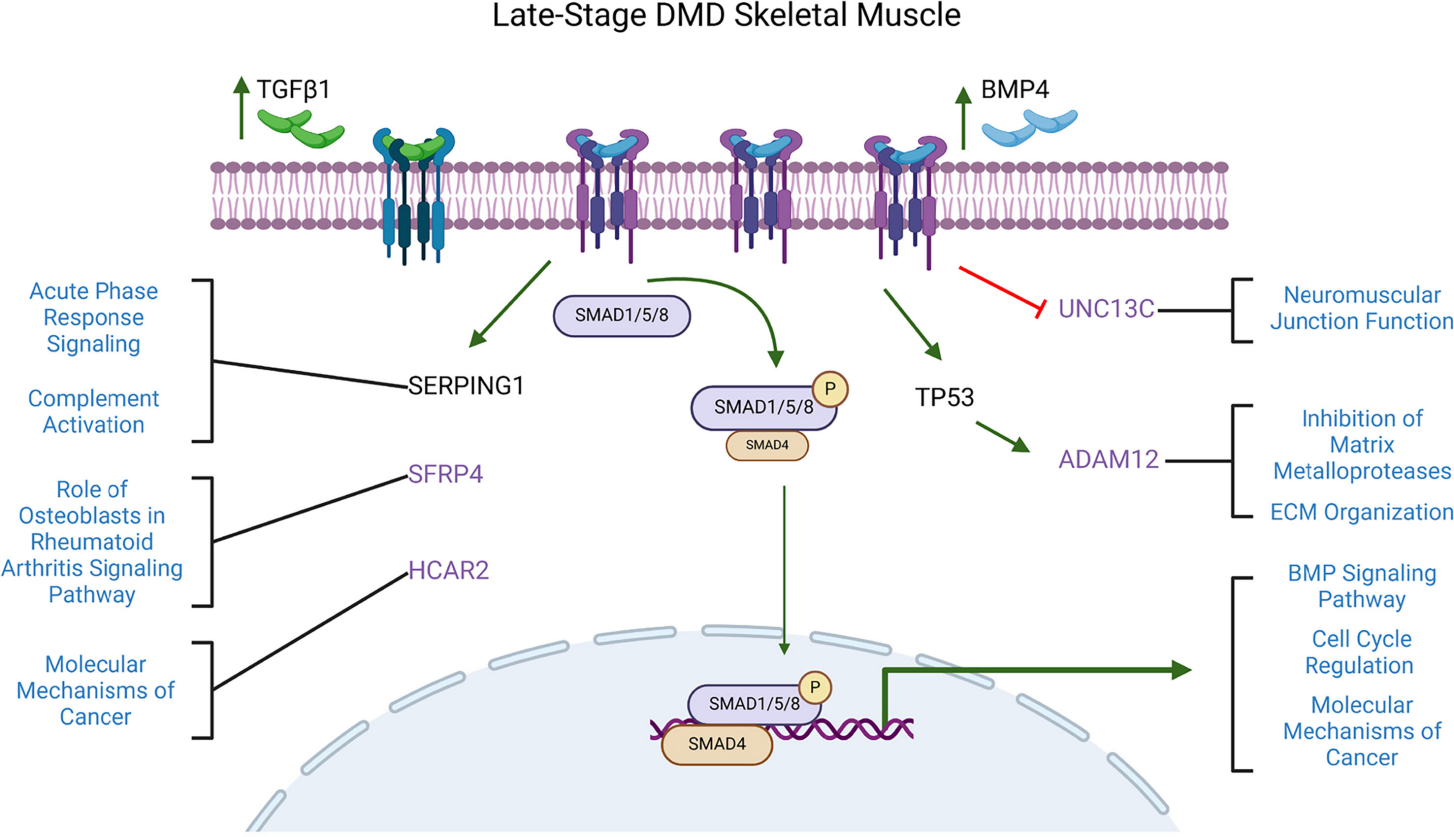
Model of late‐stage DMD skeletal muscle transcriptomic changes in TGFβ signalling pathway. Blue text indicates disease processes or canonical pathway. Purple text indicates BMP4‐induced regulation. SERPING1 and TP53 were identified as a key hub gene and an upstream regulator, respectively. Arrows and lines indicate activation (green) or inhibition (red). Illustration created in Biorender.

Module preservation analysis showed an overlapping signature of 1027 differentially upregulated transcripts (Turquoise_preserved_) in the late‐stage human DMD skeletal muscle and BMP4‐stimulated C2C12 muscle cell transcriptomes (Figure 5b and Table S18). Within this module, *SERPING1* and *Aff3* were identified as the top hub genes for the DMD and BMP4‐induced transcriptomes, respectively. Gene set enrichment analysis of this preserved transcriptomic signature identified ‘Hallmark Epithelial Mesenchymal Transition’ as the top over‐represented gene set. *ADAM12*, *SERPING1*, *SFRP4* and *SMAD8* additionally showed high‐fold upregulation by qPCR. *ADAM12* is involved in regulation of MMP and ECM organization as above. *SERPING1* is regulated by TP53, which was predicted to be an upstream regulator in DMD [37]. *SFRP4* is a WNT‐related protein involved in bone morphogenesis, WNT receptor signalling and BMP signalling [38]. It is a member of the ‘role of osteoclasts in rheumatoid arthritis signalling’ CP, which was strongly activated in both DMD and BMP4‐stimulated transcriptomes. *SMAD8*, but no other R‐SMAD, was increased in the module consistent with our prior work [8]. The overlap of BMP4‐induced transcripts with the DMD transcriptome supports this module of genes as an important signalling network involved in ECM dysregulation and which is correlated to late‐stage DMD skeletal muscle (Figure 7).

This transcriptomic study of late‐stage DMD skeletal muscle is subject to a few limitations. These include a relatively small sample size of muscle specimens and limited control over potential confounding variables such as age, sex and treatment status. Additionally, cross‐species comparisons were constrained to genes with 1:1 orthology, and even among these, functional conservation between species may be incomplete. To address these challenges, we employed multiple pathway‐ and network‐level analyses to enhance the robustness and reproducibility of our findings. Key results were further validated through qPCR and corroborated with existing literature.

Our study provides novel insight into the transcriptome of late‐stage DMD skeletal muscle and a unique in vitro BMP4‐induced transcriptomic signature. We identified common and unique transcripts, pathways and networks in late‐stage DMD from which several potential DMD biomarkers emerged. Our findings provide directions for future investigations into the role of BMP4 signalling and its downstream factors like *ADAM12*, *HCAR2*, *SMAD8*, *SFRP4* and *UNC13C*. Future investigation into BMP4/SMAD8 signalling will provide insight into the broader role of TGFβ signalling in promoting progression of DMD muscle disease.

## Conflicts of Interest

M.A.L. served as Principal Investigator on a clinical trial and participated in scientific advisory boards for Sarepta Therapeutics Inc. All other authors declare no conflicts of interest.

## Supporting information


**Figure S1** Principal component analysis (PCA) of late‐stage human DMD skeletal muscles and BMP4‐stimulated C2C12 muscle cell transcriptomes. (a) PCA plot shows the separation between the DMD and non‐DMD muscles along two dimensionless vectors PC1 and PC2. (b) PCA plot showing separation between the BMP4‐stimulated C2C12 muscle cells compared with vehicle (VEH) along two dimensionless vectors PC1 and PC2. Each group consists of 3 biological replicates (*N* = 3).

**Figure S2** Sensitivity analysis of BMP4‐stimulated C2C12 myoblast transcriptomes with and without the outlier. (a) Plot shows the log_2_ fold change (LOG_2_FC) comparing with (outlier in) and without outlier (outlier out). (b) Plot shows the adjusted (Padj) P‐Value comparing with outlier in and outlier out.

**Figure S3** Gene Set Enrichment Analysis (GSEA) of the preserved upregulated human genes (Turquoise module) overlapping in late‐stage DMD muscle and BMP4‐stimulated C2C12 muscle cells. Top gene sets showing false discovery rate (FDR), gene ratio, and gene set collection. Fill colour represents FDR. Symbol shape represents gene sets from the Molecular Signatures Database (MSigDB).

**Data S1** Supplementary Method

**Data S1** Supplementary Information.

**Table S0a** All expressed genes DMD vs. non‐DMD skeletal muscles with normalized counts.

**Table S0b** All expressed genes BMP4‐stimulated C2C12 muscle cells vs. vehicle with normalized counts.

**Table S1** Differential gene expression analysis of DMD skeletal muscles (FDR < 0.05).

**Table S2** Canonical Pathways in DMD skeletal muscle transcriptome using Ingenuity Pathway Analysis.

**Table S3** Upstream regulators in DMD skeletal muscle transcriptome using Ingenuity Pathway Analysis.

**Table S4** Analysis Match of DMD skeletal muscle transcriptome using Ingenuity Pathway Analysis.

**Table S5** Modules identified in the DMD skeletal muscle transcriptome using weighted gene co‐expression network analysis.

**Table S6** Genes in significant upregulated modules from the weighted gene co‐expression network analysis of the Duchenne muscular dystrophy skeletal muscle transcriptome

**Table S7** Genes in significant downregulated modules from the weighted gene co‐expression network analysis of the Duchenne muscular dystrophy skeletal muscle transcriptome

**Table S8** Genes highly co‐expressed with SMAD8 (based on adjacency matrix cutoff of ≥ 0.40) neighbours in turquoise module in Duchenne muscular dystrophy skeletal muscle transcriptome.

**Table S9** Genes highly co‐expressed with HCAR2 (based on adjacency matrix cutoff of ≥ 0.40) neighbours in turquoise module in Duchenne muscular dystrophy skeletal muscle transcriptome using weighted gene co‐expression network analysis.

**Table S10** Genes highly co‐expressed with DMD (based on adjacency matrix cutoff of ≥ 0.45) from the blue module in the Duchenne muscular dystrophy skeletal muscle transcriptome.

**Table S11** Differential gene expression analysis of BMP4‐stimulated C2C12 muscle cells transcriptome using DESeq method (FDR < 0.05).

**Table S12** Canonical Pathways in BMP4‐stimulated C2C12 muscle cells transcriptome using Ingenuity Pathway Analysis.

**Table S13** Upstream regulators in BMP4‐stimulated C2C12 muscle cells transcriptome using Ingenuity Pathway Analysis.

**Table S14** Modules identified in the BMP4‐stimulated C2C12 muscle cells transcriptome using weighted gene co‐expression network analysis.

**Table S15** Genes in the turquoise module of the BMP4‐stimulated C2C12 muscle cells transcriptome using weighted gene co‐expression network analysis.

**Table S16** Genes in the blue module of the BMP4‐stimulated C2C12 muscle cells transcriptome using weighted gene co‐expression network analysis.

**Table S17** Module preservation analysis between DMD muscle and BMP4‐stimulated C2C12 muscle cells transcriptomes.

**Table S18** Fold‐change of preserved genes in Turquoise modules of DMD muscle and BMP4‐ transcriptomes.

**Table S19** Gene Set Enrichment Analysis (GSEA) of preserved human DMD turquoise module. Gene set collections are from the Molecular Signatures Database (MSigDB).

**Table S20** Genes highly co‐expressed with *Unc13c* (based on adjacency matrix cutoff of ≥ 0.58) in blue module in BMP4‐stimulated C2C12 muscle cells transcriptome.
