# Supplementary figures and images for: Late‐Stage Skeletal Muscle Transcriptome in Duchenne Muscular Dystrophy Shows a BMP4‐Induced Molecular Signature
Source: J Cachexia Sarcopenia Muscle. 2025 Jul 10;16(4):e70005. doi: 10.1002/jcsm.70005 (PMC12245985; doi:10.1002/jcsm.70005)

Figure S1

a

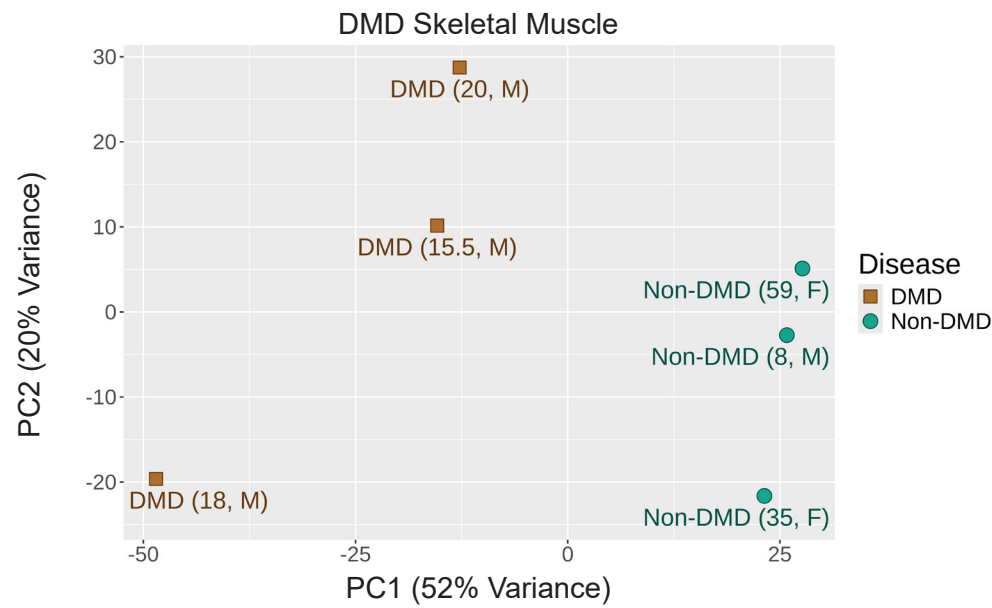

b

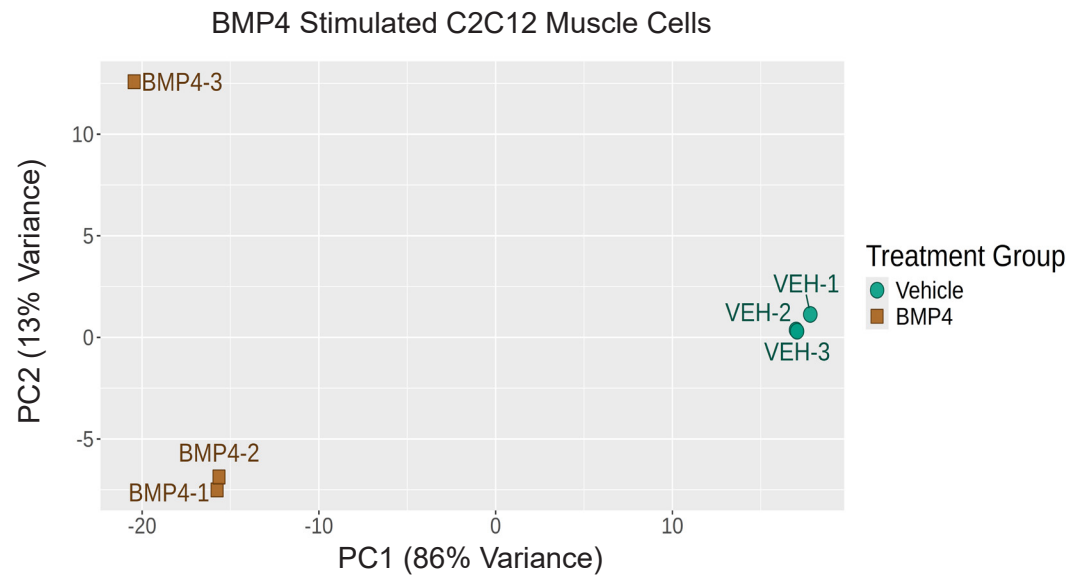

Supplement: Supplementary file 1 — Figure S1 Principal component analysis (PCA) of late‐stage human DMD skeletal muscles and BMP4‐stimulated C2C12 muscle cell transcriptomes. (a) PCA plot shows the separation between the DMD and non‐DMD muscles along two dimensionless vectors PC1 and PC2. (b) PCA plot showing separation between the BMP4‐stimulated C2C12 muscle cells compared with vehicle (VEH) along two dimensionless vectors PC1 and PC2. Each group consists of 3 biological replicates (N = 3). [file JCSM-16-e70005-s008.pdf]

Figure S2

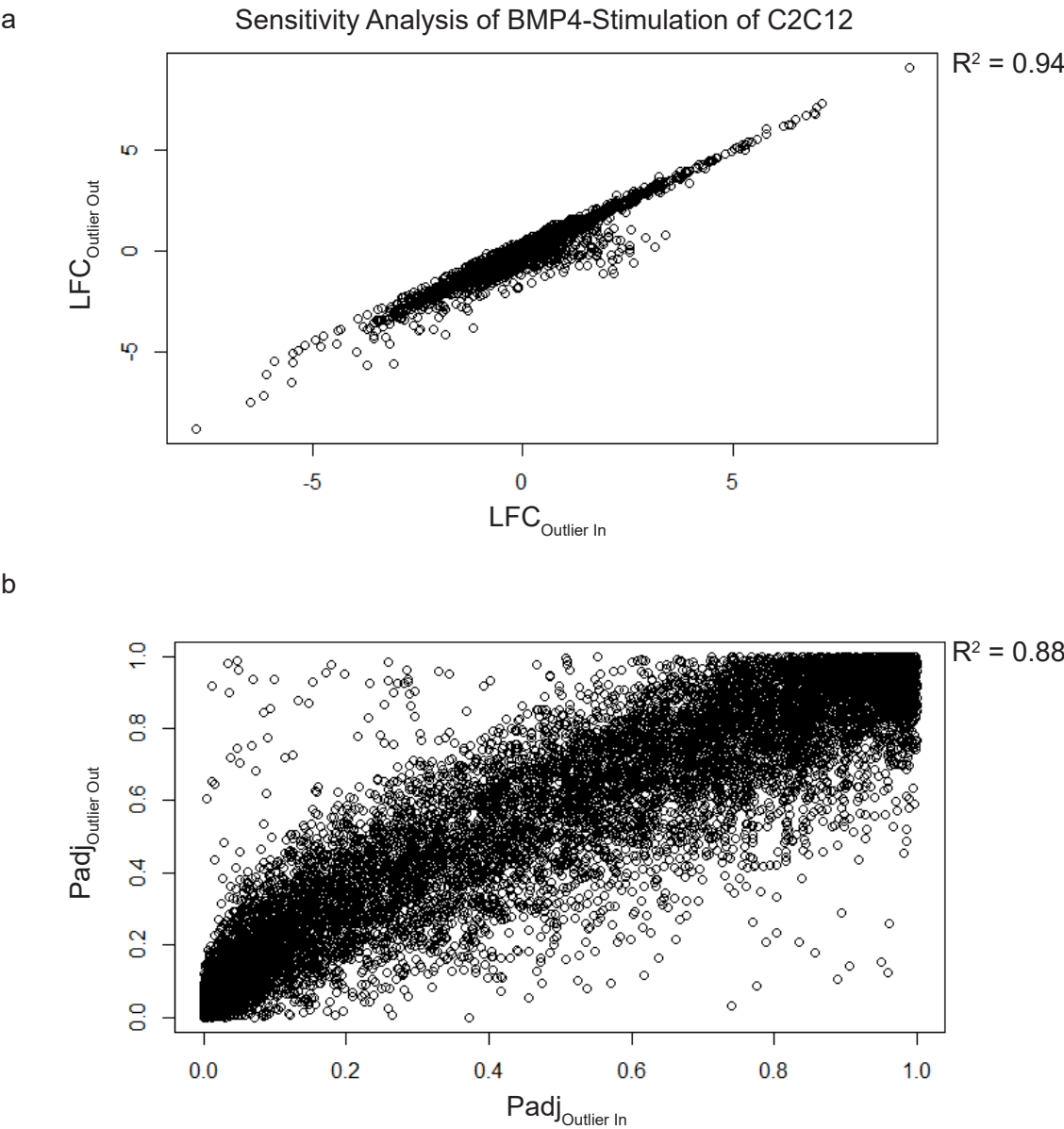

Supplement: Supplementary file 2 — Figure S2 Sensitivity analysis of BMP4‐stimulated C2C12 myoblast transcriptomes with and without the outlier. (a) Plot shows the log2 fold change (LOG2FC) comparing with (outlier in) and without outlier (outlier out). (b) Plot shows the adjusted (Padj) P‐Value comparing with outlier in and outlier out. [file JCSM-16-e70005-s001.pdf]

Figure S3

a

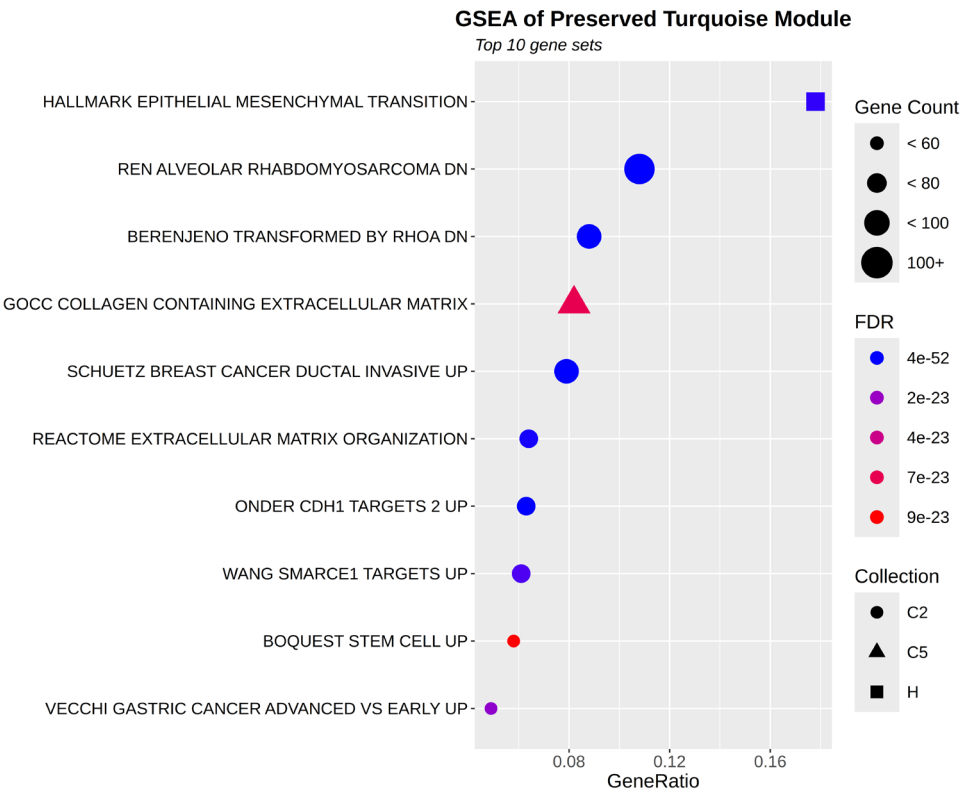

Supplement: Supplementary file 3 — Figure S3 Gene Set Enrichment Analysis (GSEA) of the preserved upregulated human genes (Turquoise module) overlapping in late‐stage DMD muscle and BMP4‐stimulated C2C12 muscle cells. Top gene sets showing false discovery rate (FDR), gene ratio, and gene set collection. Fill colour represents FDR. Symbol shape represents gene sets from the Molecular Signatures Database (MSigDB). [file JCSM-16-e70005-s007.pdf]
