# Supplementary material for: Late‐Stage Skeletal Muscle Transcriptome in Duchenne Muscular Dystrophy Shows a BMP4‐Induced Molecular Signature
Source: J Cachexia Sarcopenia Muscle. 2025 Jul 10;16(4):e70005. doi: 10.1002/jcsm.70005 (PMC12245985; doi:10.1002/jcsm.70005)
Supplement: Supplementary file 4 — Data S1 Supplementary Method [file JCSM-16-e70005-s020.pdf]

Supplemental Methods

Human Primary Myoblast Cell Isolation and Cell Culture

All normal and DMD skeletal muscle biopsies for the establishment of myoblast cell lines were obtained during either diagnostic skeletal muscle biopsies or other clinical surgeries as described previously[1, 2]. Fresh muscle biopsies were obtained, immediately dissected, digested, and filtered. Cells were plated on uncoated plates and enriched for myoblasts using pre-plating method. Myoblasts were seeded onto 6-well plates at 0.6 x 10^6 cells per well and cultured to confluency. Cells were stimulated with recombinant human BMP4 protein at 400 ng/mL for 24 hours and compared to vehicle solution (0.1% BSA, 4 mM HCL). Experiments were repeated for 3 normal and 3 DMD human muscle cell lines in triplicate. BMP4 stimulation of C2C12 muscles was performed as previously published[1].

Sequencing and RNA-Seq analysis

DNALink performed total RNA isolation using the Truseq Stranded Total RNA H/M/R Preparation Kit and Next Generation Sequencing using the Illumina NovaSeq6000 platform. Pre-processing was performed using FastQC. STAR (version 2.710a) was used to align reads to the reference genome[3]. Transcript abundances were calculated using HTSeq-count (version 2.0.2)[4]. Transcripts were aligned with all samples having > 85% uniquely mapped alignments. DESeq2 was used to calculate differential gene expression (**Table S0A, Table S0B**)[5]. Log_2 fold change (Log_2FC) and standard error show gene expression changed due to disease state or BMP4 treatment. The basic annotation performed used gene annotation from NCBI db. An outlier analysis for replicated “BMP4-3” showed no skewing of P-Values or log fold change (**Fig. S2**).

Gene Set Enrichment Analysis (GSEA)

Gene Set Enrichment Analysis (GSEA) was performed using the R packages clusterProfiler (ver. 4.4.4) and msigdbr (ver. 7.5.1). MSigDB gene sets were limited to hallmark (H), curated (C2), regulatory (C3), ontology (C5), and IMMUNESIGDB.

Gene Expression Omnibus (GEO) Search

The *Gene Expression Omnibus* (*GEO*) was queried for publicly available RNAseq datasets for DMD skeletal muscle. Search terms included: “DMD skeletal muscle”, “Duchenne Muscular Dystrophy skeletal muscle”, and “Homo sapiens [porgn: txid9606]”. Results were filtered to only show datasets with “Expression profiling by high throughput sequencing“.

Supplemental Methods References

1. Lopez, M.A., et al., *Smad8 Is Increased in Duchenne Muscular Dystrophy and Suppresses miR-1, miR-133a, and miR-133b.* International Journal of Molecular Sciences, 2022. **23**(14): p. 7515.
2. Alexander, M.S., et al., *Regulation of DMD pathology by an ankyrin-encoded miRNA.* Skelet Muscle, 2011. **1**: p. 27.
3. Dobin, A., et al., *STAR: ultrafast universal RNA-seq aligner.* Bioinformatics, 2013. **29**(1): p. 15-21.
4. Coenen-Stass, A.M.L., et al., *Comprehensive RNA-Sequencing Analysis in Serum and Muscle Reveals Novel Small RNA Signatures with Biomarker Potential for DMD.* Mol Ther Nucleic Acids, 2018. **13**: p. 1-15.
5. Love, M.I., W. Huber, and S. Anders, *Moderated estimation of fold change and dispersion for RNA-seq data with DESeq2.* Genome Biol, 2014. **15**(12): p. 550.
