# Supplementary material for: Late‐Stage Skeletal Muscle Transcriptome in Duchenne Muscular Dystrophy Shows a BMP4‐Induced Molecular Signature
Source: J Cachexia Sarcopenia Muscle. 2025 Jul 10;16(4):e70005. doi: 10.1002/jcsm.70005 (PMC12245985; doi:10.1002/jcsm.70005)
Supplement: Supplementary file 5 — Data S1 Supplementary Information. [file JCSM-16-e70005-s026.pdf]

Query:((DMD skeletal muscle) AND "Homo sapiens"[porgn:__txid9606])

Filter: Expression profiling by high throughput sequencing

Results

1. Assessment of myogenic maturation for human iPSC-derived iPAX7 myogenic progenitors in vitro and in vivo

(Submitter supplied) Here, we transplanted human iPAX7 myogenic progenitors into skeletal muscles of non-dystrophic and dystrophic mice and compared the transcriptional landscape of human donor-derived myofibers with respective in vitro-differentiated iPAX7 myotubes. Pairing bulk RNA sequencing with computational deconvolution of human reads, we were able to pinpoint key myogenic changes that occur during the in vitro-to-in vivo transition, confirm developmental maturity, and consequently further suggest the utility of cell-based therapies.

Organism: Homo sapiens; Mus musculus

Type: Expression profiling by high throughput sequencing

Platforms: GPL25526 GPL24247 GPL24676 14 Samples

FTP download: GEO (CSV) ftp://ftp.ncbi.nlm.nih.gov/geo/series/GSE244nnn/GSE244521/

Series Accession: GSE244521 ID: 200244521

2. Correction of the Exon 2, Exon 2-8 and Exons 8-9 duplications in DMD patient myogenic cells by a Single CRISPR/Cas9 system

(Submitter supplied) Duchenne Muscular dystrophy (DMD), a yet-incurable X-linked recessive disorder that results in muscle wasting and loss of ambulation is due to mutations in the dystrophin gene. Exonic duplications of dystrophin gene are a common type of mutations found in DMD patients. In this study, we utilized a single guideRNA CRISPR strategy targeting intronic regions to delete the extra duplicated regions in patient myogenic cells carrying duplication of exon 2, exons 2 to 9, and exons 8 to 9 in the DMD gene. more...

Organism: Homo sapiens

Type: Expression profiling by high throughput sequencing

Platform: GPL24676 18 Samples

FTP download: GEO (TSV, TXT) ftp://ftp.ncbi.nlm.nih.gov/geo/series/GSE248nnn/GSE248257/

Series Accession: GSE248257 ID: 200248257

3. Transcriptome profiling of human skeletal muscle organoids

(Submitter supplied) This SuperSeries is composed of the SubSeries listed below.

Organism: Homo sapiens

Type: Expression profiling by high throughput sequencing

Platforms: GPL18573 GPL21290 14 Samples

FTP download: GEO (TSV) ftp://ftp.ncbi.nlm.nih.gov/geo/series/GSE147nnn/GSE147514/

Series Accession: GSE147514 ID: 200147514

4. RNA-Seq analysis of human skeletal muscle organoids

(Submitter supplied) We performed RNA sequencing analyses of human iPSC and iPSC-derived skeletal muscle organoids in different differentiation stages of 4wk, 8wk, and 16wk.

Organism: Homo sapiens

Type: Expression profiling by high throughput sequencing

Platforms: GPL21290 GPL18573 13 Samples

FTP download: GEO (TXT) ftp://ftp.ncbi.nlm.nih.gov/geo/series/GSE147nnn/GSE147513/

SRA Run Selector: https://www.ncbi.nlm.nih.gov/Traces/study/?acc=PRJNA615097

Series Accession: GSE147513 ID: 200147513

5. Transcriptome analysis of human DMD-K2957fs and CRISPR-corrected (CORR-K2957fs) myogenic cultures during secondary myogenic differentiation.

(Submitter supplied) To investigate transcriptomes of skeletal muscle cells in health and Duchene muscular dystrophy (DMD), we performed RNA sequencing of DMD-K2957fs and CRISPR-corrected (CORR-K2957fs) myogenic cultures during secondary differentiation at 5 time points.

Organism: Homo sapiens

Type: Expression profiling by high throughput sequencing

Platform: GPL18573 40 Samples

FTP download: GEO (TAR) ftp://ftp.ncbi.nlm.nih.gov/geo/series/GSE189nnn/GSE189053/

SRA Run Selector: https://www.ncbi.nlm.nih.gov/Traces/study/?acc=PRJNA781259

Series Accession: GSE189053 ID: 200189053

6. Intron Mutations and Early Transcription Termination in Duchenne and Becker muscular dystrophy

(Submitter supplied) Purpose: DMD pathogenic variants for Duchenne and Becker muscular dystrophy are detectable with high sensitivity by standard clinical exome analyses of genomic DNA. However, up to 7% of DMD mutations are deep intronic and analysis of muscle-derived RNA is an important diagnostic step for patients who have negative genomic testing but abnormal dystrophin expression in muscle. In this study, muscle biopsies were evaluated in 19 patients with clinical features of a dystrophinopathy, but negative clinical DMD mutation analysis. more...

Organism: Homo sapiens

Type: Expression profiling by high throughput sequencing

Platform: GPL11154 11 Samples

FTP download: GEO (BW, TXT) ftp://ftp.ncbi.nlm.nih.gov/geo/series/GSE175nnn/GSE175861/

SRA Run Selector: https://www.ncbi.nlm.nih.gov/Traces/study/?acc=PRJNA734152

Series Accession: GSE175861 ID: 200175861

7. Prednisolone rescues Duchenne Muscular Dystrophy phenotypes in human pluripotent stem cells-derived skeletal muscle in vitro

(Submitter supplied) Duchenne Muscular Dystrophy (DMD) is a devastating genetic disease leading to degeneration of skeletal muscles and premature death. How dystrophin absence leads to muscle wasting remains unclear. Here, we describe an optimized protocol to differentiate human induced Pluripotent Stem Cells (iPSC) to a late myogenic stage. This allows to recapitulate classical DMD phenotypes (mislocalization of proteins of the Dystrophin glycoprotein associated complex (DGC), increased fusion, myofiber branching, force contraction defects and calcium hyperactivation) in isogenic DMD-mutant iPSC lines in vitro. more...

Organism: Homo sapiens

Type: Expression profiling by high throughput sequencing

Platform: GPL18573 45 Samples

FTP download: GEO (XLSX) ftp://ftp.ncbi.nlm.nih.gov/geo/series/GSE164nnn/GSE164874/

SRA Run Selector: https://www.ncbi.nlm.nih.gov/Traces/study/?acc=PRJNA692241

Series Accession: GSE164874 ID: 200164874

8. ERBB3 and NGFR mark distinct skeletal muscle progenitor cells in human development enabling enrichment and maturation of hPSC muscle

(Submitter supplied) Abstract: Human pluripotent stem cells (hPSCs) can be directed to differentiate into skeletal muscle progenitor cells (SMPCs). However, the myogenic potential of hPSC-SMPCs compared to human fetal or adult satellite cells (Scs) remains unclear. This study demonstrates hPSC-SMPCs derived

by commonly used protocols are functionally less mature than freshly-isolated human fetal or adult Scs. We utilized RNA-SEQ of human fetal Scs to identify differentially expressed genes including NGFR, ERBB3, which enriched for MYOD+ or PAX7+ cells. more...

Organism: Homo sapiens

Type: Expression profiling by high throughput sequencing

Platform: GPL21290 10 Samples

FTP download: GEO (XLS) ftp://ftp.ncbi.nlm.nih.gov/geo/series/GSE87nnn/GSE87365/

SRA Run Selector: https://www.ncbi.nlm.nih.gov/Traces/study/?acc=PRJNA344494

Series Accession: GSE87365 ID: 200087365

9. Gene expression profiling of human and murine in vitro muscle differentiation

(Submitter supplied) Total RNA sequencing of human and murine myoblasts and myotubes was extracted, depleted of ribosomal RNA and subjected to Illumina stranded paired end library prep and sequencing. Samples from Duchenne Muscular Dystrophy patients-derived myoblasts were included in this study

Organism: Mus musculus; Homo sapiens

Type: Expression profiling by high throughput sequencing; Non-coding RNA profiling by high throughput sequencing

Platforms: GPL11154 GPL13112 12 Samples

FTP download: GEO ftp://ftp.ncbi.nlm.nih.gov/geo/series/GSE70nnn/GSE70389/

SRA Run Selector: https://www.ncbi.nlm.nih.gov/Traces/study/?acc=PRJNA288500

Series Accession: GSE70389 ID: 200070389

10. A novel IRES identified in DMD results in a functional N-truncated dystrophin, providing a potential route to therapy for patients with 5’ mutations.

(Submitter supplied) Purpose: Ribosome profiling and RNA-Seq were used to map the location and abundance of translating ribosomes on human skeletal muscle transcripts from a patient with Becker muscular dystrophy. Methods: Tissue homogenates were prepared from frozen sections of a muscle biopsy obtained from a patient with an NM_004006:c.40_41delGA dystrophin mutation and a normal control. Ribosome-protected fragments and total RNA were prepared from a single homogenate, so starting RNA populations for both libraries were closely matched. more...

Organism: Homo sapiens

Type: Expression profiling by high throughput sequencing; Other

Platform: GPL11154 4 Samples

FTP download: GEO (TXT) ftp://ftp.ncbi.nlm.nih.gov/geo/series/GSE56nnn/GSE56148/

SRA Run Selector: https://www.ncbi.nlm.nih.gov/Traces/study/?acc=PRJNA242628

Series Accession: GSE56148 ID: 200056148

Query: ((Duchenne Muscular Dystrophy skeletal muscle) AND "Homo sapiens"[porgn:__txid9606])

Filter: Expression profiling by high throughput sequencing

Results

1. Correction of the Exon 2, Exon 2-8 and Exons 8-9 duplications in DMD patient myogenic cells by a Single CRISPR/Cas9 system

(Submitter supplied) Duchenne Muscular dystrophy (DMD), a yet-incurable X-linked recessive disorder that results in muscle wasting and loss of ambulation is due to mutations in the dystrophin gene. Exonic duplications of dystrophin gene are a common type of mutations found in DMD patients. In this study, we utilized a single guideRNA CRISPR strategy targeting intronic regions to delete the extra duplicated regions in patient myogenic cells carrying duplication of exon 2, exons 2 to 9, and exons 8 to 9 in the DMD gene. more...

Organism: Homo sapiens

Type: Expression profiling by high throughput sequencing

Platform: GPL24676 18 Samples

FTP download: GEO (TSV, TXT) ftp://ftp.ncbi.nlm.nih.gov/geo/series/GSE248nnn/GSE248257/

Series Accession: GSE248257 ID: 200248257

2. Intron Mutations and Early Transcription Termination in Duchenne and Becker muscular dystrophy

(Submitter supplied) Purpose: DMD pathogenic variants for Duchenne and Becker muscular dystrophy are detectable with high sensitivity by standard clinical exome analyses of genomic DNA. However, up to 7% of DMD mutations are deep intronic and analysis of muscle-derived RNA is an important diagnostic step for patients who have negative genomic testing but abnormal dystrophin expression in muscle. In this study, muscle biopsies were evaluated in 19 patients with clinical features of a dystrophinopathy, but negative clinical DMD mutation analysis. more...

Organism: Homo sapiens

Type: Expression profiling by high throughput sequencing

Platform: GPL11154 11 Samples

FTP download: GEO (BW, TXT) ftp://ftp.ncbi.nlm.nih.gov/geo/series/GSE175nnn/GSE175861/

SRA Run Selector: https://www.ncbi.nlm.nih.gov/Traces/study/?acc=PRJNA734152

Series Accession: GSE175861 ID: 200175861

3. Prednisolone rescues Duchenne Muscular Dystrophy phenotypes in human pluripotent stem cells-derived skeletal muscle in vitro

(Submitter supplied) Duchenne Muscular Dystrophy (DMD) is a devastating genetic disease leading to degeneration of skeletal muscles and premature death. How dystrophin absence leads to muscle wasting remains unclear. Here, we describe an optimized protocol to differentiate human induced Pluripotent Stem Cells (iPSC) to a late myogenic stage. This allows to recapitulate classical DMD phenotypes (mislocalization of proteins of the Dystrophin glycoprotein associated complex (DGC), increased fusion, myofiber branching, force contraction defects and calcium hyperactivation) in isogenic DMD-mutant iPSC lines in vitro. more...

Organism: Homo sapiens

Type: Expression profiling by high throughput sequencing

Platform: GPL18573 45 Samples

FTP download: GEO (XLSX) ftp://ftp.ncbi.nlm.nih.gov/geo/series/GSE164nnn/GSE164874/

SRA Run Selector: https://www.ncbi.nlm.nih.gov/Traces/study/?acc=PRJNA692241

Series Accession: GSE164874 ID: 200164874

4. Transcriptional profiling of Myotonic dystrophy muscle and heart

(Submitter supplied) Autopsy and biopsy muscle and heart tissue was collected from consented human subjects with and without confirmed myotonic dystrophy type 1, myotonic dystrophy type 2, or Duchenne muscular dystrophy. RNA was isolated for preparation of RNAseq libraries and sequenced on the Illumina platform.

Organism: Homo sapiens

Type: Expression profiling by high throughput sequencing

Platform: GPL11154 126 Samples

FTP download: GEO (TXT) ftp://ftp.ncbi.nlm.nih.gov/geo/series/GSE86nnn/GSE86356/

SRA Run Selector: https://www.ncbi.nlm.nih.gov/Traces/study/?acc=PRJNA342787

Series Accession: GSE86356 ID: 200086356

5. ERBB3 and NGFR mark distinct skeletal muscle progenitor cells in human development enabling enrichment and maturation of hPSC muscle

(Submitter supplied) Abstract: Human pluripotent stem cells (hPSCs) can be directed to differentiate into skeletal muscle progenitor cells (SMPCs). However, the myogenic potential of hPSC-SMPCs compared to human fetal or adult satellite cells (Scs) remains unclear. This study demonstrates hPSC-SMPCs derived by commonly used protocols are functionally less mature than freshly-isolated human fetal or adult Scs.

We utilized RNA-SEQ of human fetal Scs to identify differentially expressed genes including NGFR, ERBB3, which enriched for MYOD+ or PAX7+ cells. more...

Organism: Homo sapiens

Type: Expression profiling by high throughput sequencing

Platform: GPL21290 10 Samples

FTP download: GEO (XLS) ftp://ftp.ncbi.nlm.nih.gov/geo/series/GSE87nnn/GSE87365/

SRA Run Selector: https://www.ncbi.nlm.nih.gov/Traces/study/?acc=PRJNA344494

Series Accession: GSE87365 ID: 200087365

6. Gene expression profiling of human and murine in vitro muscle differentiation

(Submitter supplied) Total RNA sequencing of human and murine myoblasts and myotubes was extracted, depleted of ribosomal RNA and subjected to Illumina stranded paired end library prep and sequencing. Samples from Duchenne Muscular Dystrophy patients-derived myoblasts were included in this study

Organism: Mus musculus; Homo sapiens

Type: Expression profiling by high throughput sequencing; Non-coding RNA profiling by high throughput sequencing

Platforms: GPL11154 GPL13112 12 Samples

FTP download: GEO ftp://ftp.ncbi.nlm.nih.gov/geo/series/GSE70nnn/GSE70389/

SRA Run Selector: https://www.ncbi.nlm.nih.gov/Traces/study/?acc=PRJNA288500

Series Accession: GSE70389 ID: 200070389

7. A novel IRES identified in DMD results in a functional N-truncated dystrophin, providing a potential route to therapy for patients with 5’ mutations.

(Submitter supplied) Purpose: Ribosome profiling and RNA-Seq were used to map the location and abundance of translating ribosomes on human skeletal muscle transcripts from a patient with Becker muscular dystrophy. Methods: Tissue homogenates were prepared from frozen sections of a muscle biopsy obtained from a patient with an NM_004006:c.40_41delGA dystrophin mutation and a normal control. Ribosome-protected fragments and total RNA were prepared from a single homogenate, so starting RNA populations for both libraries were closely matched. more...

Organism: Homo sapiens

Type: Expression profiling by high throughput sequencing; Other

Platform: GPL11154 4 Samples

FTP download: GEO (TXT) ftp://ftp.ncbi.nlm.nih.gov/geo/series/GSE56nnn/GSE56148/

SRA Run Selector: https://www.ncbi.nlm.nih.gov/Traces/study/?acc=PRJNA242628

Series Accession: GSE56148 ID: 200056148
